# An Improved Vision Method for Robust Monitoring of Multi-Point Dynamic Displacements with Smartphones in an Interference Environment

**DOI:** 10.3390/s20205929

**Published:** 2020-10-20

**Authors:** Taicong Chen, Zhou Zhou

**Affiliations:** 1 School of Civil Engineering and Transportation, South China University of Technology, Guangzhou 510641, China; ctzhouzhou@mail.scut.edu.cn; 2 State Key Laboratory of Subtropical Building Science, South China University of Technology, Guangzhou 510641, China

**Keywords:** computer vision, multi-point displacement measurement, motion enhanced spatio-temporal context, optical flow

## Abstract

Current research on dynamic displacement measurement based on computer vision mostly requires professional high-speed cameras and an ideal shooting environment to ensure the performance and accuracy of the analysis. However, the high cost of the camera and strict requirements of sharp image contrast and stable environment during the shooting process limit the broad application of the technology. This paper proposes an improved vision method to implement multi-point dynamic displacement measurements with smartphones in an interference environment. A motion-enhanced spatio-temporal context (MSTC) algorithm is developed and applied together with the optical flow (OF) algorithm to realize a simultaneous tracking and dynamic displacement extraction of multiple points on a vibrating structure in the interference environment. Finally, a sine-sweep vibration experiment on a cantilever sphere model is presented to validate the feasibility of the proposed method in a wide-band frequency range. In the test, a smartphone was used to shoot the vibration process of the sine-sweep-excited sphere, and illumination change, fog interference, and camera jitter were artificially simulated to represent the interference environment. The results of the proposed method are compared to conventional displacement sensor data and current vision method results. It is demonstrated that, in an interference environment, (1) the OF method is prone to mismatch the feature points and leads to data deviated or lost; (2) the conventional STC method is sensitive to target selection and can effectively track those targets having a large proportion of pixels in the context with motion tendency similar to the target center; (3) the proposed MSTC method, however, can ease the sensitivity to target selection through in-depth processing of the information in the context and finally enhance the robustness of the target tracking. In addition, the MSTC method takes less than one second to track each target between adjacent frame images, implying a potential for online measurement.

## 1. Introduction

Structural health monitoring, as a crucial technique ensuring long-term structural safety, plays an essential role in early warning, evaluation, and maintenance decisions during the structural life cycle. Accelerometers, one kind of data sensors widely used in structural health monitoring practices, can directly measure the acceleration response of structures while less effective in the low-frequency range [1]. In contrast, displacement sensors can obtain sensitive low-frequency dynamic measurements but see relatively few applications due to the inconvenience of installation and monitoring. For instance, a linear variable differential transformer (LVDT) requires a fixed reference point to measure the displacement, and the laser Doppler vibrometer needs installing close to the object.

In recent years, with the development of image data acquisition and computer vision techniques, vision-based displacement measurement technology has attracted extensive research attention due to its advantages of no contact, long distance, and low cost [2,3]. According to target markers required or not, the computer vision technologies for displacement measurement can be classified into two categories. The first category using customized target markers [4,5,6,7,8,9,10,11] can increase the differentiation between the tracking target and the background to improve measurement accuracy. In the actual measurement, however, the installation of markers is labor-intensive, especially when multi-point displacements are desired.

In contrast, the non-target measurement category uses the texture features of the structure surface as pseudo markers. Various methods have been developed to track the features points and identify the corresponding displacements. For instance, with feature-point matching algorithms, Khuc [12] and Dong [13] adopted professional high-speed cameras to achieve displacement measurement without attaching any artificial target markers. This kind of algorithms mostly needs a clear texture of the object to reduce the false recognition of features. Moreover, the use of high-speed cameras also requires high-brightness lighting conditions to achieve a sharp contrast of imaging objects. Yoon [14] applied an optical flow algorithm and adopted a consumer-grade camera to extract the displacement response and dynamic characteristics. Feng [15] proposed a sub-pixel template matching technique based on image correlation, improving the accuracy of displacement extraction. It is worth noting that all the above studies are applied under ideal measurement conditions and require a stable lighting environment. The influence of unfavorable environmental factors such as illumination change, fog interference, camera motion, and complex background on the measurement results is not involved. Regarding this, Dong [16] applied a spatio-temporal context (STC) algorithm to implement the displacement measurement with a high-speed camera in the illumination change and fog environment. The research realized single-point dynamic displacement measurement but not multi-point synchronous monitoring that is more favorable in structural health monitoring practice. Furthermore, the applied STC algorithm treats the whole region of the context equally, which weakens the effectiveness of the context information. As for the camera motion problem, which is getting increasing attention in vision measurement practices, a general solution is to compensate for camera motion-induced errors by subtracting the movement of reference points on the background [17,18]. The methods applied therein to identify the reference-point movement are critical to the measurement accuracy, especially when camera jitter, one particular kind of camera motion undergoing dramatic change and hence increasing challenges, is taken into account.

The present study proposed an improved displacement measurement method with computer vision technology, which allows the use of smartphone-recording video to achieve simultaneous monitoring of multi-point dynamic displacements under changing environmental conditions. It is noted that current smartphones with high-resolution cameras preserve many advantages, such as low cost, portability, and ease of use, making it a competitive device for optical sensing under various conditions [19]. In the proposed method, after calibration of the sequential distorted images shot from the smartphone, multiple areas centered at the targeting points are predefined with permissible overlapping. A motion-enhanced spatio-temporal context (MSTC) algorithm is then developed for robust tracking of the moving targets. Therein, a motion-enhanced function is constructed with an optical flow (OF) algorithm to strengthen the beneficial information and weaken the interference information in the whole context area. Finally, based on the tracked pixel-level results from the MSTC algorithm, the OF algorithm is re-applied to estimate the subpixel-level moving, benefiting eventually to the achievement of high-precision displacement results. To validate the feasibility of the proposed method for monitoring vibrating displacements in a wide frequency range, a sine-sweep vibration experiment on a cantilever sphere model is carried out. In the test, an iPhone 7 was used to shoot the vibration process of the sine-sweep-excited sphere, and illumination change, fog interference, and camera jitter were artificially simulated to represent the interference environment. By referring to the displacement sensor data, comparative studies between results from the proposed method and current vision methods were presented.

## 2. Integrated Process for Multi-Point Displacement Measurement

Implementation of the proposed measurement method focuses on three aspects: smartphone application, multi-point monitoring, and high-precision displacement extraction.

The first aspect needs to solve the calibration of the wide-angle distortion from the consumer-grade camera of the smartphone. Therein, Zhang’s method [20] is employed to implement the camera calibration, and we further propose an iterative calibration procedure based on a continually expanding image library to achieve calibrated parameters converged.

The second aspect concerns the issue of multi-target selection and definition. Therein, through introducing multi-region compatibility and parallel design, we extend the range of the STC algorithm from conventional single-target tracking to multi-target tracking.

The last aspect deals with the algorithms about multi-target tracking and high-precision displacement extraction under environmental interference, which is the most crucial part of the integrated process. Therein, we develop a new tracking algorithm, i.e., the MSTC algorithm, to implement robust tracking of multiple objects. A sub-pixel displacement estimation method [21] is then employed to refine the measurement.

**Figure 1:**
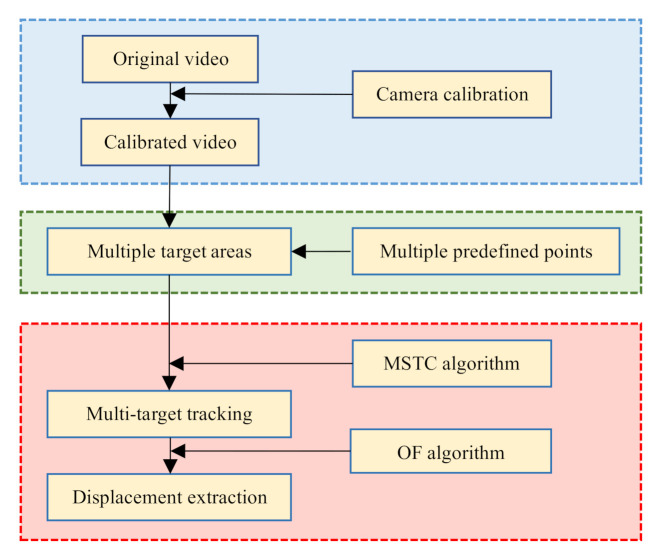
Flowchart of multi-displacement measurement with smartphone.

Figure 1 shows the flowchart of the monitoring process, in which three main parts are integrated with sequence and will be illustrated in detail in the following.

## 3. Smartphone-Shoot Image Calibration

In most smartphones, the consumer-grade cameras are equipped with a wide-angle lens to increase the shooting range, while it will introduce large radial and tangential distortion to the image. Therefore, to reduce the position error caused by the image distortion, it is necessary to calibrate the camera and identify the internal/external parameters and distortion coefficients.

According to the pinhole model used for camera imaging [20], the relationship between a real point *A* (*X*, *Y*, *Z*) and the corresponding point *A*’(*u*, *v*) in the camera image can be described as

(1) $$s\left[\begin{array}{ccc} x & y & 1 \end{array}\right]=\left[\begin{array}{cccc} X & Y & Z & 1 \end{array}\right]\left[\begin{array}{c} R\\ t \end{array}\right]$$

(2) $$\left[\begin{array}{ccc} u & v & 1 \end{array}\right]=\left[\begin{array}{ccc} x & y & 1 \end{array}\right]K$$

where (*X*, *Y*, *Z*), (*x*, *y*), and (*u*, *v*) represent the coordinates of point *A* in the real-world coordinate system, camera coordinate system, and pixel coordinate system, respectively; *s* is a scaling factor; the external parameter matrices *R* and *t,* respectively, represent the rotation matrix and the translation matrix, related to the physical position of the camera; the internal parameter matrix *K* is determined by the camera structure and material, represented as

(3) $$K=\;\left[\begin{array}{ccc} f_{x} & 0 & 0\\ \theta & f_{y} & 0\\ u_{0} & v_{0} & 1 \end{array}\right]$$

where $f_{x}$ and $f_{y}$ are scale factors of the *x* and *y* axes in the pixel coordinate system; θ is the inclination parameter of the axis; $u_{0}$ and $v_{0}$ are coordinates of the origin.

Equations (1) and (2) represent an ideal imaging relationship. However, the camera lens produces radial and tangential distortions, causing the imaging point to shift. A nonlinear distortion model [22] can be used to describe the distortions as

(4) $$\left[\begin{array}{c} x_{e}\\ y_{e} \end{array}\right]=\left(1+k_{1}r^{2}+k_{2}r^{4}\right)\left[\begin{array}{c} x_{d}\\ y_{d} \end{array}\right]\;+\left[\begin{array}{c} 2p_{1}x_{d}y_{d}+p_{2}\left(r^{2}+2x_{d}^{2}\right)\\ 2p_{2}x_{d}y_{d}+p_{1}\left(r^{2}+2y_{d}^{2}\right) \end{array}\right]$$

in which ($x_{e}$, $y_{e}$) and ($x_{d}$, $y_{d}$) are the ideal exact coordinates and actual distorted coordinates of the target point in the camera coordinate system, respectively; $k_{1}$ and $k_{2}$ are radial distortion coefficients; $p_{1}$ and $p_{2}$ are tangential distortion coefficients; *r* is a radius defined by

(5) $$r^{2}=x_{d}^{2}+y_{d}^{2}$$

Identification of internal/external parameters and distortion coefficients, namely, camera calibration, is based on the accurate coordinates (*X*, *Y*, *Z*) of several predefined points and the distorted pixel coordinates ($x_{d}$, $y_{d}$) of the corresponding imaging points. Specific values of all the parameters and coefficients can be solved by minimizing the sum of square errors between the distorted pixel coordinates ($x_{d}$, $y_{d}$) and the pixel coordinates projected from (*X*, *Y*, *Z*) with Equations (1)–(5).

**Figure 2:**
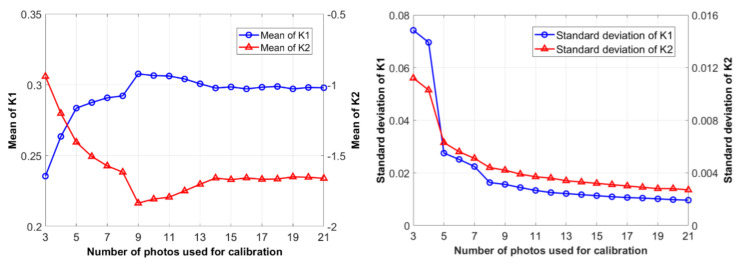
Identification trend of radial distortion coefficients.

In the present study, we use the method proposed in Reference [20] to implement the camera calibration. Firstly, an image database is established by taking photos at a predefined chessboard with the smartphone, an iPhone 7, of different locations and postures. Then a series of calibration analyses adopting various numbers of images are carried out until a converged result is reached, as listed in Table 1 and Table 2 for final results of all the parameters and coefficients of the iPhone 7 rear camera. Figure 2 shows the variation of identified coefficients $k_{1}$ and $k_{2}$ versus the number of images used. It can be observed that nearly 20 images are sufficient to meet a convergence requirement for the camera calibration.

**Figure 3:**
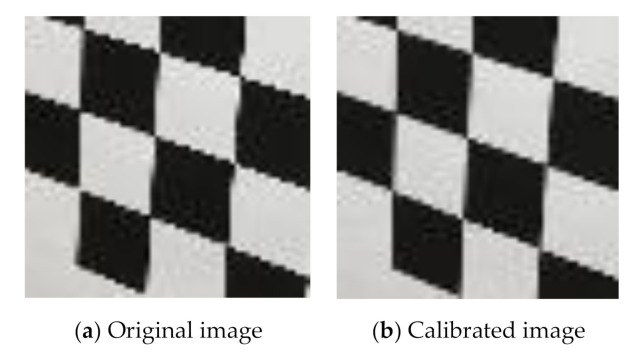
Pre- and post-calibration images of B&W chessboard.

After the camera calibration, we apply Equation (4) for every interested image to adjust the distorted pixel coordinates ($x_{d}$,$y_{d}$) to a more rational one $\left(x_{c},y_{c}\right)$, which is finally used in Equations (1) and (2) to get the real-world coordinates. Figure 3 shows a comparison of a partial image of the chessboard before and after calibration. It can be seen in the original image that, due to radial and tangential distortions, vertical edges of black and white squares are projected as curves and fail to form a straight line, especially for the most marginal black square in the bottom left corner. At the same time, apparent zigzag happens on horizontal edges. In the calibrated image, the distorted vertical edges have been recovered nearly straight, and the zigzagged horizontal edges have been partly smoothed. The calibration, although not 100% recovery, still favors in an error reduction of the final displacement.

## 4. Multi-Target Definition

In the structural vibration practice, multi-point responses are substantially desired for a comprehensive understanding of vibration and consequent modal analysis. By use of video-based methods to achieve multi-point displacement measurement, one needs to make a clear definition of all the points and track their positions in each frame image of the video.

**Figure 4:**
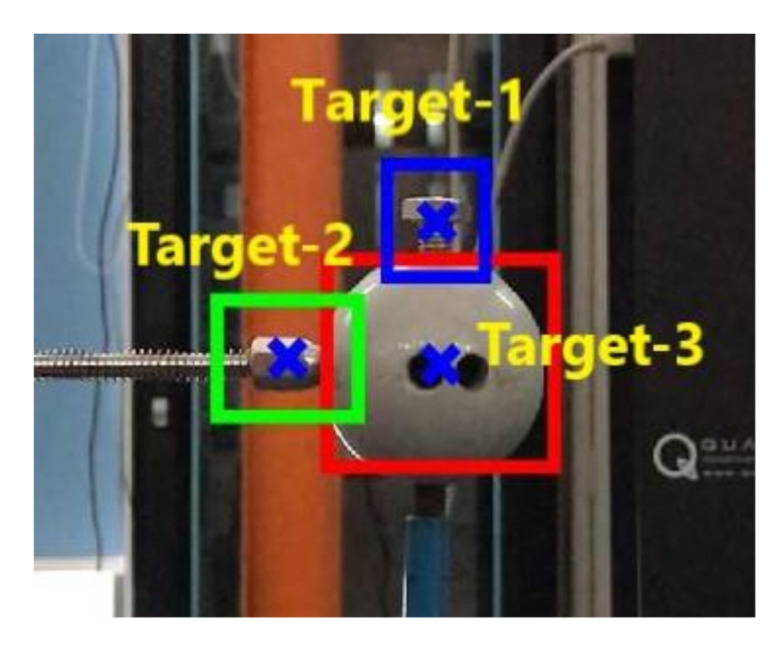
Definition of three target areas in the first frame image.

The STC method used in the present study was initially developed to monitor a single-target movement [16,23]. Herein, we extend its application range to the multi-target case by introducing the regional compatibility of multiple target areas into the parallel design of the target-tracking algorithm. The definition of three target areas in the first frame image is shown in Figure 4. Each area is of a rectangle shape and centers at a predefined target point of cross mark. It is worth noting that the target areas allow partially overlapped, and the number of targets is indeed unlimited. Movements of the corresponding target areas then represent those of the target points.

## 5. Target Tracking Based on MSTC Algorithm

### 5.1. Conventional STC Tracking Algorithm

The STC algorithm formulates the spatio-temporal statistical correlation between the image intensity from the object of interest and its local context and obtains the best target position by maximizing the following confidence function [23],

(6) $$c\left(x\right)=P\left(x|o\right)=ae^{-{\left(\frac{\left|x-x^{*}\right|}{\alpha }\right)}^{\beta }}$$

where $x\in R^{2}$ is the target position; o denotes the target present in the scene; $x^{*}$ is the center point of the target area; a is a normalization constant; α and β are a scale parameter and a shape parameter, respectively.

**Figure 5:**
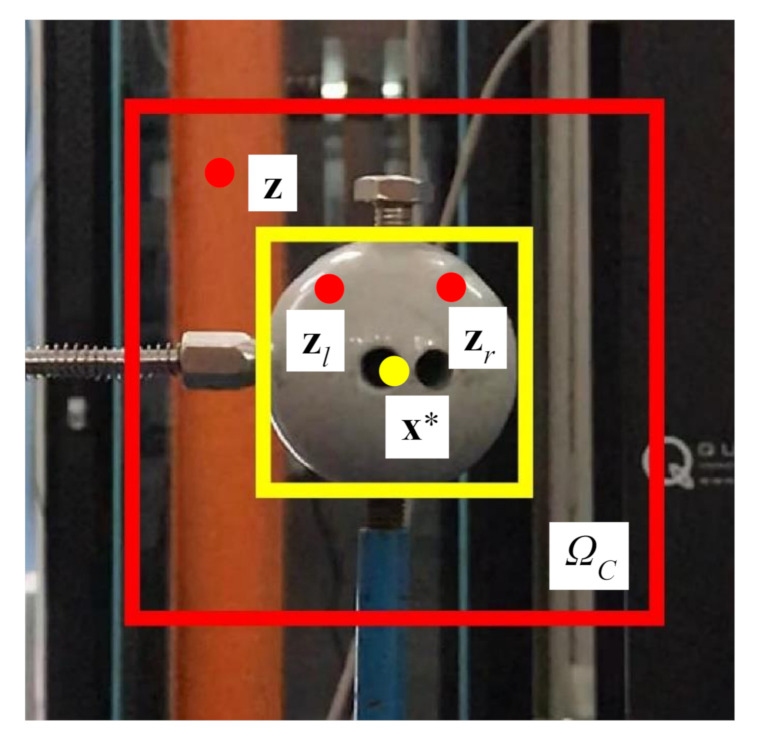
Graphical model of the spatial relationship between the target and its local context.

In a current frame image, the context feature set is defined as,

(7) $$X^{c}=\left\{c\left(z\right)=\left(I\left(z\right),z\right)|z\in \Omega_{C}\left(x^{*}\right)\right\}$$

where $I\left(z\right)$ denotes image intensity at location z; $\Omega_{C}\left(x^{*}\right)$ is the context area around the target. As shown in Figure 5, the yellow box defines the tracking target, and the red box represents its local context.

According to the total probability formula, the confidence function in Equation (6) can be computed by

(8) $$c\left(x\right)=P\left(x|o\right)=\sum_{c\left(z\right)\in X^{c}}P\left(x,c\left(z\right)|o\right)\;=\sum_{c\left(z\right)\in X^{c}}P\left(x|c\left(z\right),o\right)P\left(c\left(z\right)|o\right)$$

where, $P\left(x,c\left(z\right)|o\right)$ is the joint probability; $P\left(x|c\left(z\right),o\right)$ is the conditional probability, modeling the spatial relationship between the target and its context;$\;P\left(c\left(z\right)|o\right)$ is the context prior probability, modeling the appearance characteristics of the context. The main task in STC is to learn $P\left(x|c\left(z\right),o\right)$ as bridging the target position and its spatial context.

With the intensity of the current frame image, the context prior probability $P\left(c\left(z\right)|o\right)$ is simply modeled as

(9) $$P\left(c\left(z\right)|o\right)=I\left(z\right)\omega_{\sigma }\left(z-x^{*}\right)$$

where

(10) $$\omega_{\sigma }\left(z-x^{*}\right)=\;be^{-\frac{{\left|z-x^{*}\right|}^{2}}{\sigma^{2}}}$$

(11) $$\sigma =\left(s_{w}+s_{h}\right)/2$$

in which, b is a normalization constant; σ is a scale parameter; $s_{w}$ and $s_{h}$ are the length and width of the target area, respectively.

The conditional probability $P\left(x|c\left(z\right),o\right)$ is modeled to be a function of the relative distance and direction between the target and its context,

(12) $$P\left(x|c\left(z\right),o\right)=h\left(x-z\right)$$

Substituting Equations (9) and (12) into Equation (8), we can get

(13) $$c\left(x\right)=\sum_{c\left(z\right)\in X^{c}}h\left(x-z\right)I\left(z\right)\omega_{\sigma }\left(z-x^{*}\right)=h\left(x\right)⊗\left(I\left(x\right)\omega_{\sigma }\left(x-x^{*}\right)\right)$$

where ⊗ denotes the convolution operator. For the sake of efficient operation, Equation (13) is transformed into the frequency domain so that the Fast Fourier Transform (FFT) algorithm can be used for fast convolution, leading to

(14) $$\;\;F\left(c\left(x\right)\right)=F\left(h\left(x\right)\right)⊙F\left(I\left(x\right)\omega_{\sigma }\left(x-x^{*}\right)\right)\;$$

where F denotes the FFT operation and ⊙ represents the pixel-wise product. Therefore, we have

(15) $$h\left(x\right)=F^{-1}\left(\frac{F\left(c\left(x\right)\right)}{F\left(I\left(x\right)\omega_{\sigma }\left(x-x^{*}\right)\right)}\right)=F^{-1}\left(\frac{F\left(ae^{-{\left(\frac{\left|x-x^{*}\right|}{\alpha }\right)}^{\beta }}\right)}{F\left(I\left(x\right)\omega_{\sigma }\left(x-x^{*}\right)\right)}\right)\;$$

where $F^{-1}\;$denotes the inverse FFT operation.

After $h\left(x\right)$ is obtained, the tracking problem with STC is simply formulated as a detection task. In the (*t* + 1)-th frame image, the object position $x_{t+1}^{*}$ is determined by maximizing the new confidence function

(16) $$x_{t+1}^{*}=\underset{x\in \Omega_{C}\left(x_{t}^{*}\right)}{argmax}\;c_{t+1}\left(x\right)$$

where $c_{t+1}\left(x\right)$ is represented as

(17) $$c_{t+1}\left(x\right)=F^{-1}\left(F\left(H_{t+1}\left(x\right)\right)⊙F\left(I_{t+1}\left(x\right)\omega_{\sigma_{t}}\left(x-x^{*}\right)\right)\right)$$

in which $H_{t+1}\left(x\right)$, a replacement of $h\left(x\right)$ in Equation (14) with additional temporal information, is updated by

(18) $$H_{t+1}\left(x\right)\;=\left(1-\rho \right)H_{t}\left(x\right)+\rho h_{t}\left(x\right)$$

where ρ is a learning rate factor.

### 5.2. Motion-Enhanced STC (MSTC) Tracking Algorithm

#### 5.2.1. Basic Idea

With the spatio-temporal context model in Equation (18), the conventional STC algorithm can filter out the image noise caused by appearance variations, thereby leading to stable tracking results. However, the STC algorithm treats the entire context equivalently by taking it for granted that all regions of the context have the same contribution in tracking the target movement, leading to ineffective outcomes in a severe noise environment. Regarding this shortcoming, Xu [24] proposed a weighted STC algorithm by applying Harris corner displacement identification and linear interpolation to construct a weight function. However, the Harris corner tracking may fail in an interference environment, as shown in Section 7, and the linear interpolation with a few feature points can only yield an approximate contribution of the context.

**Figure 6:**
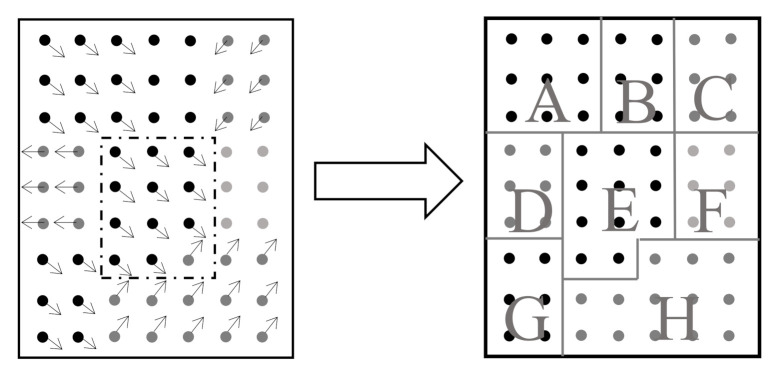
Context and division result.

We notice in practical videos that pixels on the same moving object tend to have a more similar motion than the other pixels, implying different pixels in the context may have different correlations with the target in terms of motion tendency. An example of the pixel-wise motion tendency obtained from the optical flow (OF) algorithm (details in Section 5.2.2) is shown in Figure 6. Regarding the similarity with the target center, all pixels in the context can be roughly grouped into three sets: (1) the auxiliary set, e.g., regions A, E, and G; (2) the irrelevant set, e.g., regions B and F; (3) the interference set, e.g., regions C, D, and H. Furthermore, in line with varying movements in the video process, these three sets will also vary frame to frame during the tracking process. Such new spatio-temporal information, if integrated into the tracking algorithm, will benefit a lot to improve the measurement accuracy in a noisy environment.

Therefore, the basic idea of the MSTC algorithm we develop here is to construct a dense pixel-wise influence matrix by using the motion-tendency similarity between pixels in the context and the target. Pixels in the regions of the auxiliary set will have the most substantial influence on target tracking, as opposed to the interference set. The influence matrix is then integrated into the tracking framework of the STC algorithm to achieve more robust tracking performance.

#### 5.2.2. Derivation of the Context Influence Matrix

The context influence matrix builds a mathematical model based on the motion-tendency similarity between pixels in different regions of the context and the tracking target. Here, we apply the OF approach to identify the motion tendency of all pixels in the context to generate the context influence matrix. The procedure is as follows:

(1) Get the intensity $I_{t-1}\left(x,y\right)$ of the (*t* − 1)-th frame image and $I_{t}\left(x,y\right)$ of the *t*-th frame image.
(2) Identify the pixel-wise velocity by use of the least-square OF method [25],
  (19) $$\left[\begin{array}{cc} \underset{x,y}{\sum }{\left(\frac{\partial I_{t}}{\partial x}\right)}^{2}\; & \underset{x,y}{\sum }\frac{\partial I_{t}}{\partial x}\frac{\partial I_{t}}{\partial y}\\ \underset{x,y}{\sum }\frac{\partial I_{t}}{\partial x}\frac{\partial I_{t}}{\partial y} & \underset{x,y}{\sum }{\left(\frac{\partial I_{t}}{\partial y}\right)}^{2}\; \end{array}\right]\left[\begin{array}{c} {\left(v_{x}\right)}_{t}\\ {\left(v_{y}\right)}_{t} \end{array}\right]=\;\left[\begin{array}{c} \underset{x,y}{\sum }\frac{\partial I_{t}}{\partial t}\frac{\partial I_{t}}{\partial x}\\ \underset{x,y}{\sum }\frac{\partial I_{t}}{\partial t}\frac{\partial I_{t}}{\partial y} \end{array}\right]$$
  where all the partial derivatives can be estimated by averaging the first-order finite differential values, i.e.,
  (20) $$\;\frac{\partial I_{t}}{\partial x}\approx \frac{1}{4}\left(I_{t-1}\left(x+1,y\right)+I_{t-1}\left(x+1,y+1\right)+I_{t}\left(x+1,y\right)+I_{t}\left(x+1,y+1\right)\right)\;-\frac{1}{4}\left(I_{t-1}\left(x,y\right)+I_{t-1}\left(x,y+1\right)+I_{t}\left(x,y\right)+I_{t}\left(x,y+1\right)\right)$$
  (21) $$\frac{\partial I_{t}}{\partial y}\approx \frac{1}{4}\left(I_{t-1}\left(x,y+1\right)+I_{t-1}\left(x+1,y+1\right)+I_{t}\left(x,y+1\right)+I_{t}\left(x+1,y+1\right)\right)\;-\frac{1}{4}\left(I_{t-1}\left(x,y\right)+I_{t-1}\left(x+1,y\right)+I_{t}\left(x,y\right)+I_{t}\left(x+1,y\right)\right).\;$$
  (22) $$\;\frac{\partial I_{t}}{\partial t}\approx \frac{1}{4}\left(I_{t}\left(x,y\right)+I_{t}\left(x+1,y\right)+I_{t}\left(x,y+1\right)+I_{t}\left(x+1,y+1\right)\right)\;-\frac{1}{4}\left(I_{t-1}\left(x,y\right)+I_{t-1}\left(x+1,y\right)+I_{t-1}\left(x,y+1\right)+I_{t-1}\left(x+1,y+1\right)\right)$$
(3) Calculate the velocity difference between the target center and each pixel in the context,
  (23) $$\;d_{t}^{i}=\sqrt{{\left({\left(v_{x}^{*}\right)}_{t}-{\left(v_{x}^{i}\right)}_{t}\right)}^{2}+{\left({\left(v_{y}^{*}\right)}_{t}-{\left(v_{y}^{i}\right)}_{t}\right)}^{2}}$$
(4) For each pixel in the context, convert the corresponding velocity difference with a negative exponential function to generate the influence coefficient,
  (24) $$m_{t}^{i}=e^{-\frac{{\left(d_{t}^{i}\right)}^{2}}{2\gamma^{2}}}$$
  where γ is a scale parameter. As can be seen that more similar the two velocities are, i.e., less velocity difference, the more considerable value the influence coefficient will take.

**Figure 7:**
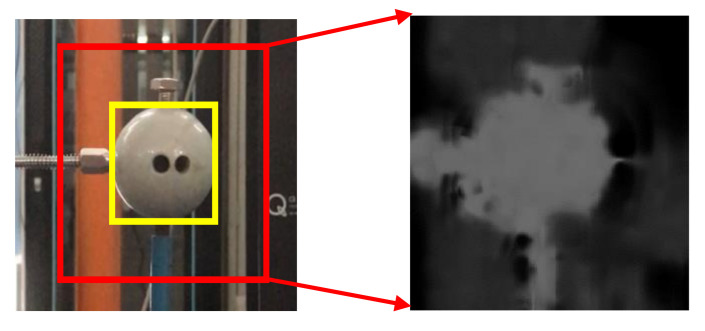
Local context and influence matrix.

As shown in Figure 7 is an example of the influence matrix, which we obtained in the laboratory verification for tracking Target Point 2 under fog interference (Scenario III, see details in Section 7). The bright region in the context is almost consistent with the profile of the moving object, implying that a correct relationship is successfully built between the target center and the pixels in the context. Such new information helps to include more contributions from the context into the tracking of the target, especially when severe interference is involved.

#### 5.2.3. Updated Formulation

By combining the influence of the context, we renew the conditional probability as

(25) $$P\left(x|c\left(z\right),o\right)=h\left(x-z\right)*m\left(z\right)$$

where $m\left(z\right)$ is the dense pixel-wise influence matrix.

Correspondingly, all the related formulas are updated as

(26) $$c\left(x\right)=\sum_{c\left(z\right)\in X^{c}}h\left(x-z\right)m\left(z\right)I\left(z\right)\omega_{\sigma }\left(z-x^{*}\right)=h\left(x\right)⊗\left(m\left(x\right)I\left(x\right)\omega_{\sigma }\left(x-x^{*}\right)\right)$$

(27) $$\;\;F\left(c\left(x\right)\right)=F\left(h\left(x\right)\right)⊙F\left(m\left(x\right)I\left(x\right)\omega_{\sigma }\left(x-x^{*}\right)\right)\;$$

(28) $$h\left(x\right)=F^{-1}\left(\frac{F\left(c\left(x\right)\right)}{F\left(m\left(x\right)I\left(x\right)\omega_{\sigma }\left(x-x^{*}\right)\right)}\right)=F^{-1}\left(\frac{F\left(ae^{-{\left(\frac{\left|x-x^{*}\right|}{\alpha }\right)}^{\beta }}\right)}{F\left(m\left(x\right)I\left(x\right)\omega_{\sigma }\left(x-x^{*}\right)\right)}\right)\;.\;$$

#### 5.2.4. MSTC Tracking Process

After $m\left(x\right)$ is obtained, the confidence function to be maximized during the tracking process now becomes

(29) $$c_{t+1}\left(x\right)=F^{-1}\left(F\left(H_{t+1}\left(x\right)\right)⊙F\left(M_{t+1}\left(x\right)I_{t+1}\left(x\right)\omega_{\sigma_{t}}\left(x-x^{*}\right)\right)\right).\;$$

in which $M_{t+1}\left(x\right)$, a temporal version of $m\left(x\right)$ and similar to $H_{t+1}\left(x\right)$ in Equation (18), is updated by

(30) $$M_{t+1}\left(x\right)\;=\left(1-\eta \right)M_{t}\left(x\right)\;+\;\eta m_{t}\left(x\right)$$

where η is a learning rate factor and the initial matrix $M_{1}$ is defined as a regularized matrix with all entries equal to 1.

**Figure 8:**
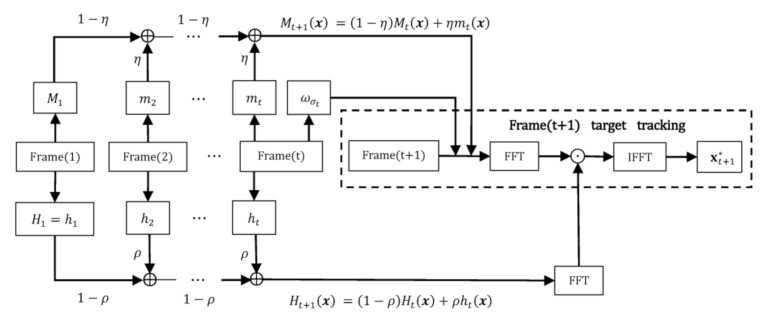
Flow chart of motion-enhanced spatio-temporal context (MSTC) algorithm.

The integrated MSTC tracking process is illustrated in Figure 8. For the sake of a robust tracking, we recommend several crucial parameters taking values as, α = 2.25, β = 1, ρ = 0.075, γ = 4, η = 0.3.

## 6. Sub-Pixel Displacement Estimation with OF Algorithm

The target position obtained above is of integer pixel, resulting in a pixel-level displacement. To achieve more accurate movement, besides choosing a higher-resolution camera, one can also use the OF algorithm to estimate the sub-pixel displacement [21].

In general, the following relationship holds between the target intensities in two adjacent frame images, e.g., $I_{t-1}\left(x,y\right)$ and $I_{t}\left(x,y\right)$,

(31) $$I_{t-1}\left(x,y\right)=I_{t}\left(x+\Delta x,y+\Delta y\right)$$

where the target displacement $\left(\Delta x,\;\Delta y\right)$ can be expressed as the sum of the pixel-level part $\left(\bar{\Delta x},\;\bar{\Delta y}\right)$ and the sub-pixel part $\left(\delta x,\;\delta y\right)$

(32) $$\left\{\begin{array}{c} \Delta x=\bar{\Delta x}+\delta x\\ \Delta y=\bar{\Delta y}+\delta y \end{array}\right.$$

in which δx≪1 and δy≪1.

After the pixel-level displacement $\left(\bar{\Delta x},\;\bar{\Delta y}\right)$ has been identified by using the MSTC algorithm, we can construct a new template image relating to the pixel-level displacement, then apply the OF algorithm to expand the $I_{t-1}\left(x,y\right)$ in terms of δx and δy,

(33) $$I_{t-1}\left(x,y\right)\;=\;I_{t}\left(x+\bar{\Delta x}+\delta x,y+\bar{\Delta y}+\delta y\right)\approx {\bar{I}}_{t}\left(x,y\right)+\frac{\partial {\bar{I}}_{t}\left(x,y\right)}{\partial x}\delta x+\frac{\partial {\bar{I}}_{t}\left(x,y\right)}{\partial y}\delta y$$

where ${\bar{I}}_{t}\left(x,y\right)=I_{t}\left(x+\bar{\Delta x},y+\bar{\Delta y}\right)$ represents the intensity of the new template image.

Then, by applying the same least-square OF method as in Section 5.2.2, we can get

(34) $$\left[\begin{array}{cc} \underset{x,y}{\sum }{\left(\frac{\partial {\bar{I}}_{t}}{\partial x}\right)}^{2}\; & \underset{x,y}{\sum }\frac{\partial {\bar{I}}_{t}}{\partial x}\frac{\partial {\bar{I}}_{t}}{\partial y}\\ \underset{x,y}{\sum }\frac{\partial {\bar{I}}_{t}}{\partial x}\frac{\partial {\bar{I}}_{t}}{\partial y} & \underset{x,y}{\sum }{\left(\frac{\partial {\bar{I}}_{t}}{\partial y}\right)}^{2}\; \end{array}\right]\left[\begin{array}{c} \delta x\\ \delta y \end{array}\right]=\;\left[\begin{array}{c} \underset{x,y}{\sum }\left(I_{t-1}-{\bar{I}}_{t}\right)\frac{\partial {\bar{I}}_{t}}{\partial x}\\ \underset{x,y}{\sum }\left(I_{t-1}-{\bar{I}}_{t}\right)\frac{\partial {\bar{I}}_{t}}{\partial y} \end{array}\right]$$

where all the partial derivative can be estimated similarly as in Section 5.2.2.

Related research [21] has shown that the accuracy of sub-pixel displacement estimated by the OF algorithm can reach as small as 0.0125 pixel. Therefore, the combination of the MSTC tracking algorithm to identify the pixel-level movement and the OF algorithm to estimate the sub-pixel displacement can effectively improve the measurement precision of the target displacement. It is also worth noting that the same OF algorithm is adopted in the present study to estimate both the pixel-wise motion tendency and the sub-pixel displacement, facilitating the implementation of compact and unified programming.

## 7. Laboratory Verification

In the following, we will carry out forced vibration experiments of a cantilever sphere model to verify whether the proposed method can accurately obtain the multi-point displacement time history of the model subject to light change, fog interference, and camera jitter. Based on smartphone-shoot videos recording the model vibration process, measurement results with the proposed MSTC method are compared to the displacement sensor data, as well as those obtained with two current vision methods, e.g., the conventional STC method [16] and the characteristic OF method [14].

### 7.1. Experiment Setup

**Figure 9:**
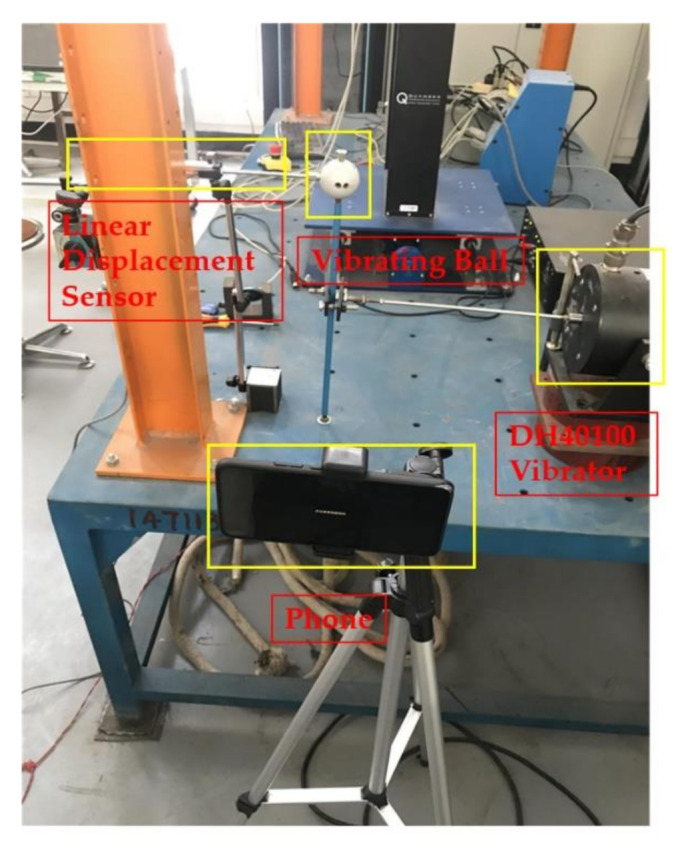
Experiment layout.

Figure 9 shows the experiment layout. A steel sphere is installed on top of a vertical steel rod that is bottom-fixed on a rigid support and connected on the middle to a vibration exciter, which will input external excitation to the structural model and force the sphere to vibrate horizontally. A linear variable differential transformer (LVDT), in which one end connected to the sphere and the other end fixed to the left steel column, is used to measure the displacement of the sphere, with data automatically collected by a computer. An iPhone7 smartphone mounted on a tripod in front of the model is used to shoot the sphere vibration video. The resolution of the iPhone7 rear camera is set to 1920 × 1080 pixels and the theoretical frame rate set to 60 frames per second (FPS). The actual frame rate of the video, however, is 59.96 FPS. This subtle FPS difference may cause significant errors to the displacement result, as will be illustrated in detail later. It should also be noted that we deliberately keep the complex background, without cover like usual doing, to approach the real application environment.

**Figure 10:**
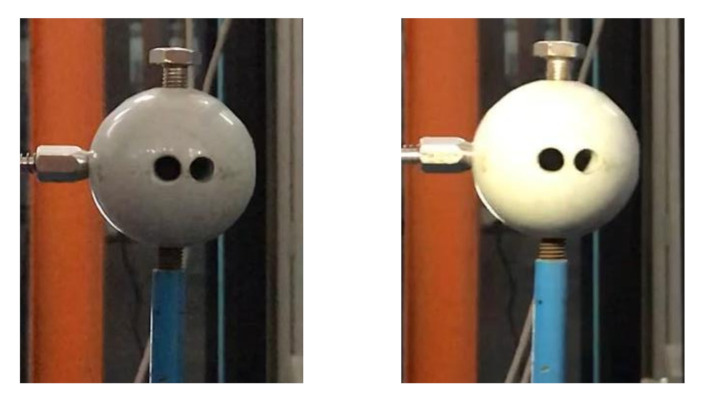
Comparison of variable illumination.

**Figure 11:**
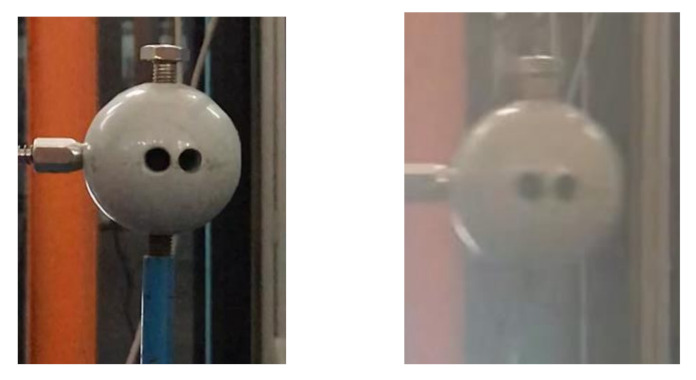
Comparison of fog simulation.

**Figure 12:**
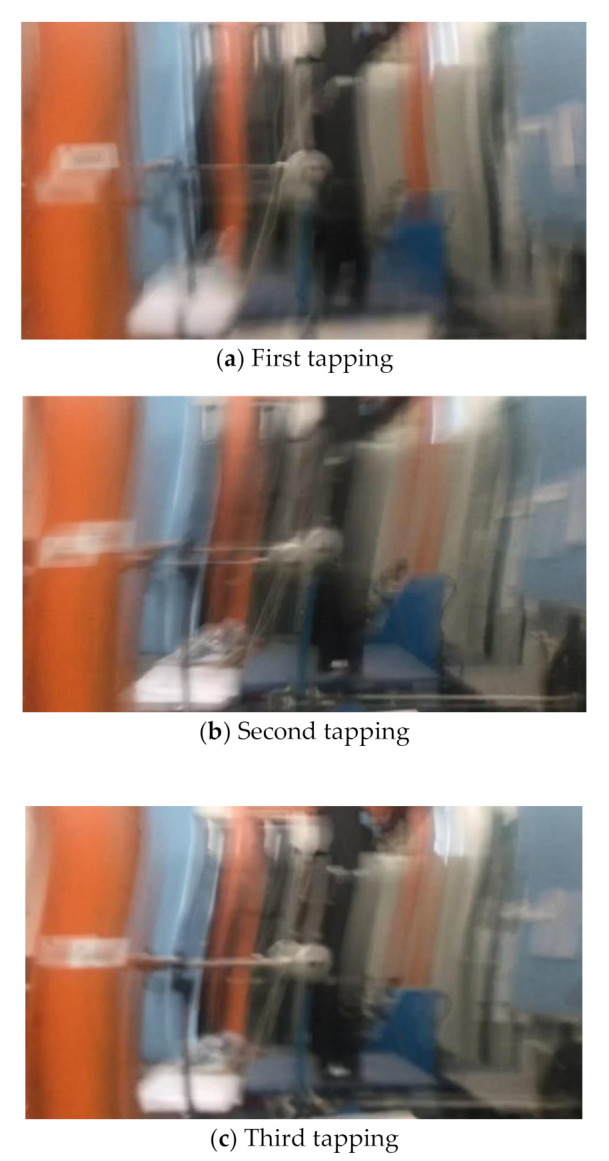
Blurred images after each tapping.

**Figure 13:**
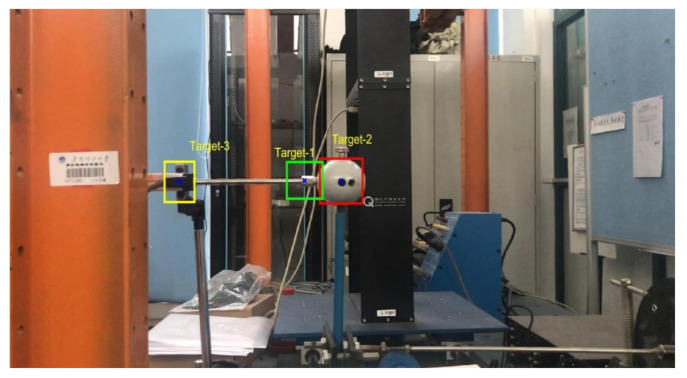
Initial definition of three tracking targets.

In the experiment, for verification in a wide-band frequency range, the exciter is set to produce repetitive sine-sweep excitation with frequency linearly varying from 1 Hz to 10 Hz. We design four scenarios to examine the application effect of the vision methods in different environments,

(1) Scenario I: no interference in the environment during the vibration.
(2) Scenario II: illumination variation in the environment during the vibration. We place a lamp near the sphere and switch it on/off several times during the vibration. As shown in Figure 10, the left image represents the case when the lamp is switched off, and the right image when switched on. A significant difference in image brightness can be observed between the two cases.
(3) Scenario III: fog interference in the environment during the vibration. We place a humidifier between the sphere and the smartphone and turn it on during the vibration to simulate the foggy environment. As shown in Figure 11, the left image represents the case when the humidifier is turned off and the right image when turned on. A significant difference in image contrast can be observed between the two cases.
(4) Scenario IV: camera jitter during the vibration. We use a pencil to tap the tripod three times during the vibration to make the camera jitter happen after each tapping. As shown in Figure 12 are typical frame images taken from the video at the time right after each tapping. A comparison between Figure 12 and Figure 13 (see later for non-vibrating state) shows that the images are severely blurred due to the camera jitter, in which one can hardly find clear features points.

### 7.2. Measurement Results

We define three points as the target points that locates at a similar height as the measured point of the LVDT. As shown in Figure 13 are the definition, in the first frame image, of the three target points denoted with blue cross marks and the corresponding target areas indicated with green, red, and yellow boxes. It can be seen that the first target point is sitting on the tip end of the LVDT, the second one is nearly the center of the sphere, and the third one is on the fixed end of the LVDT. During the vibration, the third target point is indeed non-moving. Therefore, in Scenarios I-III, we only track the first and second target points. Moreover, in Scenario IV, we further track the third target point for the sake of excluding the camera-jitter influence from the measurements of the first and second target points.

We firstly adjust the original vibration video shot by the smartphone to a new one by using all the calibrated parameters and coefficients obtained in Section 3, then apply three vision-based measurement methods, i.e., MSTC, STC, and characteristic OF, to the new video to get the corresponding time–history displacements. The characteristic OF method [14] applied herein uses a tracking algorithm that combines Lucas-Kanade optical flow and Harris corners, in which the points for tracking cannot be artificially defined but automatically determined with critical features in the first frame image. So, the tracked points with this OF method cannot be exactly the ones defined above for the MSTC and STC, and we choose one of those feature points that is nearest to the second target point for comparative study.

In the comparative study, we use two types of evaluation indicators [26], e.g., the normalized mean square error indicator (*NMSE*) and the correlation coefficient indicator (ρ), by referring to the LVDT data to evaluate the overall performances of the vision-based measurement methods,

(35) $$NMSE=1-\frac{\sum_{i}^{n}{\left(d_{v}\left(i\right)-d_{s}\left(i\right)\right)}^{2}}{\sum_{i}^{n}{\left(d_{s}\left(i\right)-\bar{d_{s}}\right)}^{2}}$$

(36) $$\rho =\frac{\left|\sum_{i=1}^{n}\left(d_{v}\left(i\right)-\bar{d_{v}}\right)\left(d_{s}\left(i\right)-\bar{d_{s}}\right)\right|}{\sqrt{\sum_{i=1}^{n}{\left(d_{v}\left(i\right)-\bar{d_{v}}\right)}^{2}\cdot \sum_{i=1}^{n}{\left(d_{s}\left(i\right)-\bar{d_{s}}\right)}^{2}}}$$

where, $d_{v}\left(i\right)$ and $d_{s}\left(i\right)$ are the *i*-th time-instant displacements obtained with the vision method and the LVDT, respectively; $\bar{d_{v}}$ and $\bar{d_{s}}$ represent the corresponding mean values of the time–history displacements. For both indicators, a value closer to 1 indicates a better agreement between the vision-measured result and the LVDT data. It is noted that, besides these two overall indicators, some local indices such as deviations from maximum or minimum values are also investigated in the present study for comprehensive understanding.

**Figure 14:**
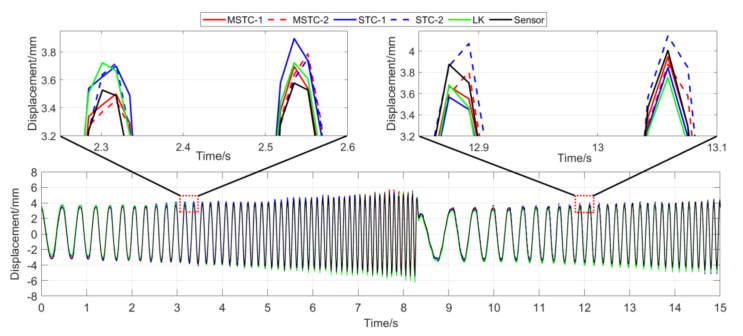
Displacement time history and local amplification in Scenario I (59.96 FPS).

**Figure 15:**
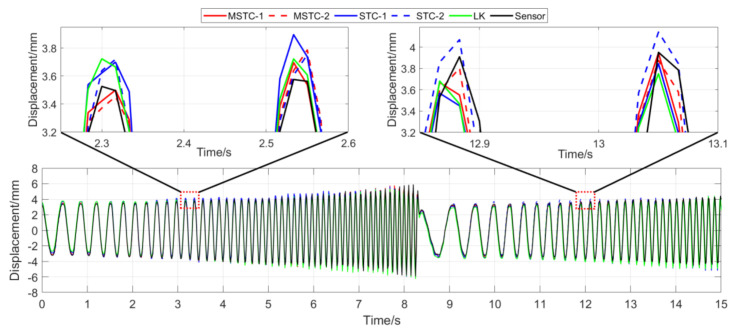
Displacement time history and local amplification in Scenario I (60 FPS).

**Figure 16:**
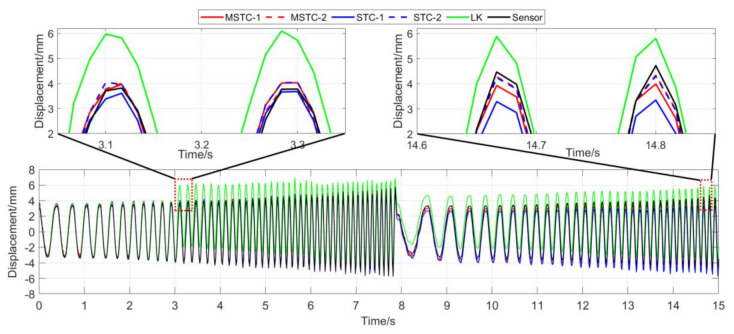
Displacement time history and local amplification in Scenario II (59.96 FPS).

**Figure 17:**
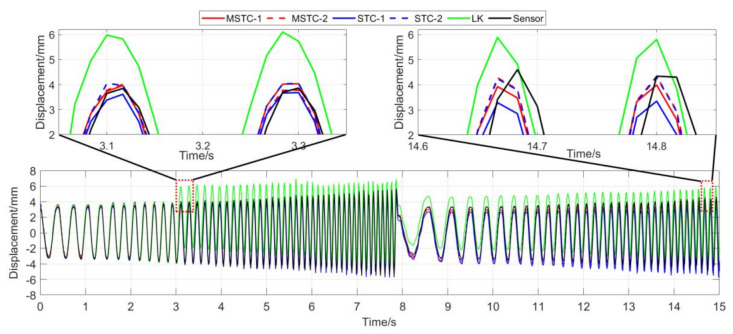
Displacement time history and local amplification in Scenario II (60 FPS).

**Figure 18:**
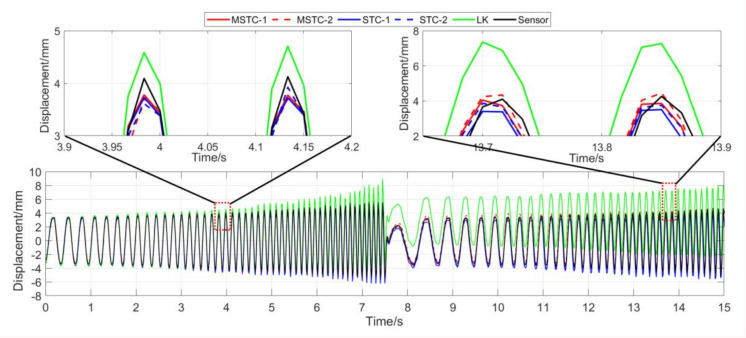
Displacement time history and local amplification in Scenario III (59.96 FPS).

**Figure 19:**
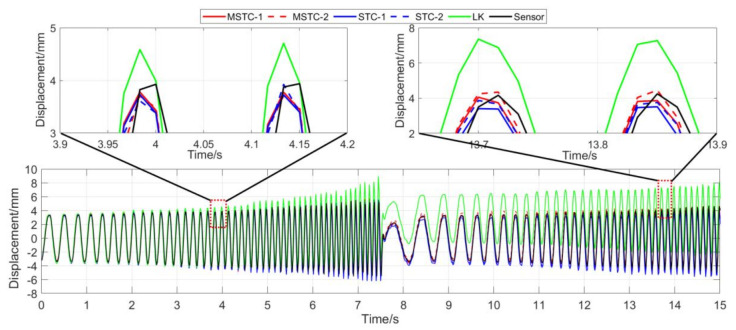
Displacement time history and local amplification in Scenario III (60 FPS).

**Figure 20:**
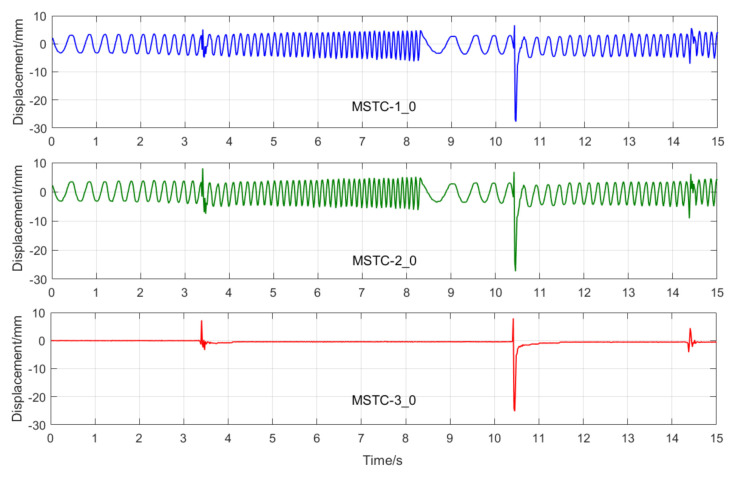

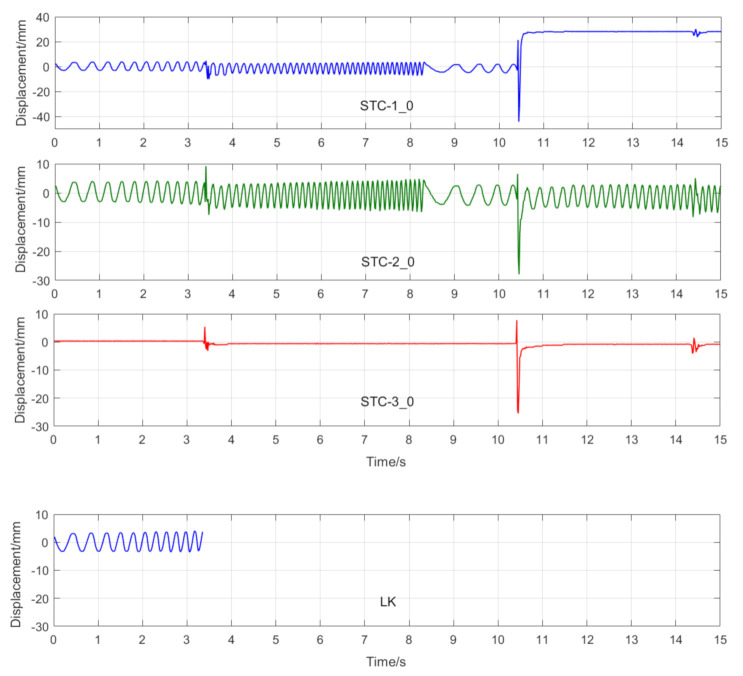
Overall measurement time history and local amplification in Scenario IV.

**Figure 21:**
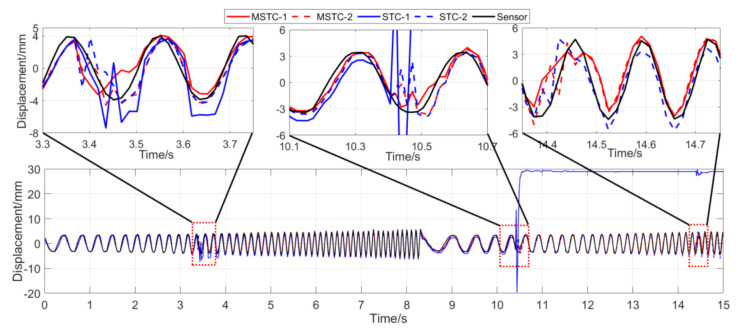
Displacement time history and local amplification in Scenario IV (59.96 FPS).

**Figure 22:**
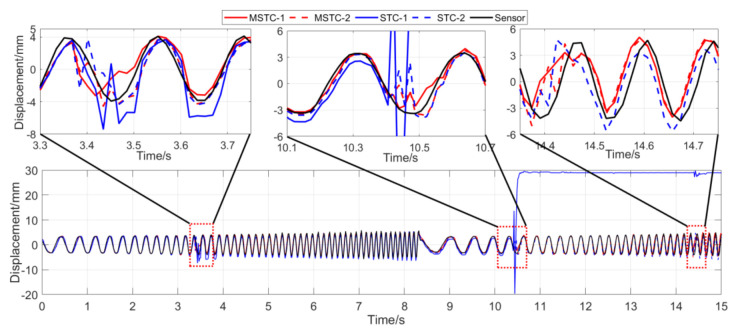
Displacement time history and local amplification in Scenario IV (60 FPS).

With the first 15-s time history, the final measurement results in all four scenarios are shown and compared in Figure 14, Figure 15, Figure 16, Figure 17, Figure 18, Figure 19, Figure 20, Figure 21 and Figure 22 and Table 3, Table 4, Table 5 and Table 6. Therein, MSTC-1 and MSTC-2 denote the results of the first target point and the second target point identified by the MSTC method, respectively; STC-1 and STC-2 denote the ones by the STC method; LK denotes the ones by the characteristic OF method; sensor denotes the LVDT data. It is noted that the sampling rate for the LVDT is 100 Hz and it is down-sampled to be comparable to the vision measurements with the theoretical or actual frame rate [27].

In Scenario IV, MSTC-1_0, MSTC-2_0, and MSTC-3_0 denote the overall results of the three target points identified by the MSTC method, which include the camera-jitter influence. Corresponding displacements of the first and second target points, denoted by MSTC-1 and MSTC-2, respectively, are then obtained by excluding the camera-jitter influence, e.g., subtracting MSTC-3_0 from MSTC-1_0 and MSTC-2_0. With the STC method, similar definitions hold for STC-1_0, STC-2_0, STC-3_0, STC-1, and STC-2.

Observations on the results will be illustrated in the following.

#### 7.2.1. Scenario I

From Figure 14 and Figure 15 and Table 3, it can be seen that,

(1) In the absence of interference factors, all three vision methods can accurately capture the sphere’s movement under the complex background, among which the two STC-type methods perform better than the OF method, and the MSTC method provides the most consistent results with the LVDT data.
(2) Both overall indicators, i.e., *NMSE* and ρ, can reach the same conclusion in terms of distinguishing the advantages and disadvantages of different methods, and *NMSE* shows higher sensitivity. See the 59.95 FPS case, for example, ρ keeps larger than 0.99 with all methods while *NMSE* takes 0.992, 0.988, and 0.987 for MSTC-1, STC-1, and LK, respectively.
(3) The measurement results obtained with the actual frame rate (59.96 FPS) are more accurate than the ones with the theoretical frame rate (60 FPS). The inconsistency of the frame rate of smartphones between theory and practice has been noticed by Yoon [14], and our study further shows that corresponding errors in displacement measurement even deteriorate in a frequency-varying vibration practice. In the theoretical FPS case, significant shifting happens during the whole time history and exaggerates in later-stage vibration due to error accumulation. Variations of overall indicators also demonstrate the influence. See the MSTC-1 measurement, for example, due to inadequate choice of 60 FPS instead of 59.96 FPS, *NMSE* drops from 0.992 to 0.953 and ρ from 0.996 to 0.977.
(4) The maximum and minimum values from the sensor vary with different frame rates due to the down-sampled operation. The corresponding deviations, although not wholly accurate, can still provide an estimation of the local accuracy. For the MSTC method, deviation results are relatively small, e.g., less than 10%, implying a good accuracy of the proposed method.

#### 7.2.2. Scenario II

From Figure 16 and Figure 17 and Table 4, it can be seen that,

(1) When the illumination undergoes a sudden change during the vibration, the displacement time history obtained with the OF method varies abruptly, indicating failure of the target tracking. However, results with the STC-type methods still keep stable and consistent with the LVDT data in shape and trend. Besides, again, the MSTC method gives the best performance.
(2) Both overall indicators can lead to the same conclusion in comparing different methods’ performance under illumination variation, and, again, *NMSE* is more sensitive. See the 59.95 FPS case, for example, ρ keeps larger than 0.96 with all methods, while *NMSE* takes 0.977, 0.957, and 0.778 for MSTC-1, STC-1, and LK, respectively. Besides, when compared with Scenario I, *NMSE* drops from 0.987 to 0.778 for LK, from 0.988 to 0.957 for STC-1, and from 0.992 to 0.977 for MSTC-1. The minimum loss of *NMSE* indicates the best performance of the MSTC method in resisting the interference of illumination variation.
(3) A significant influence on the measurement results similar to Scenario I still holds for the frame rate of the smartphone. See the MSTC-1 measurement, for example, due to an inadequate choice of 60 FPS instead of 59.96 FPS, *NMSE* drops from 0.977 to 0.885 and ρ from 0.989 to 0.945.
(4) The MSTC method and the STC method perform almost the same in measuring target 2, while the former one shows better performance in measuring target 1. See also in Table 4, *NMSE* takes 0.988 and 0.987 for MSTC-2 and STC-2, respectively, while 0.977 and 0.957 for MSTC-1 and STC-1, respectively. As shown in Figure 13, the proportion of pixels in the target-1 context with similar motion tendency to the target center is much smaller than that in the target-2 one, implying fewer auxiliary information and more interference one for target 1. It results in a more difficult tracking of target 1 under an interference environment. At the same time, the MSTC method can achieve better indices than the STC method by extracting the favorable and unfavorable information from the context and correspondingly strengthening or weakening its influence during the target tracking.
(5) The most significant deviations happen to the OF method. Moreover, deviations of the MSTC method are smaller than those of the STC method, in which the target 2 shows better performance than the target 1.

#### 7.2.3. Scenario III

From Figure 18 and Figure 19 and Table 5, it can be seen that,

(1) When the fog continuously interferes with the shooting environment during the vibration, the displacement time history obtained with the OF method significantly shifts from the accurate one, indicating apparent failure of the target tracking. However, the same excellent performance of the MSTC method and the STC method, as in Scenario II, still holds.
(2) Both overall indicators can lead to the same conclusion in comparing different methods’ performance under fog interference, and, again, *NMSE* is more sensitive. See the 59.95 FPS case, for example, ρ keeps larger than 0.91 with all methods while *NMSE* takes 0.969, 0.948, and 0.501 for MSTC-1, STC-1, and LK, respectively. Besides, when compared with Scenario I, *NMSE* drops from 0.987 to 0.501 for LK, from 0.988 to 0.948 for STC-1, and from 0.992 to 0.969 for MSTC-1. The minimum loss of *NMSE* indicates the best performance of the MSTC method in resisting the fog interference.
(3) A significant influence on the measurement results similar to Scenarios I and II still holds for the frame rate of the smartphone. See the MSTC-1 measurement, for example, due to an inadequate choice of 60 FPS instead of 59.96 FPS, *NMSE* drops from 0.969 to 0.872 and ρ from 0.986 to 0.939.
(4) The MSTC method, again, shows similar performance as the STC method in measuring target 2 and better performance in measuring target 1, indicating a better anti-interference ability of the proposed method. See also in Table 5, *NMSE* takes 0.972 and 0.971 for MSTC-2 and STC-2, respectively, while 0.969 and 0.948 for MSTC-1 and STC-1, respectively.
(5) The most significant deviations happen to the OF method. Moreover, deviations of the MSTC method are smaller than those of the STC method.

#### 7.2.4. Scenario IV

From Figure 20, Figure 21 and Figure 22 and Table 6, it can be seen that,

(1) When camera jitter happens during the vibration, the OF method fails to find and match the critical feature points in the blurred images. The STC method may significantly mistrack the target point when subjected to substantial camera jitter, as shown in STC-1_0 after the second tapping. The MSTC method, however, can deliver excellent performance in tracking all three target points under all camera-jitter circumstances.
(2) Both overall indicators can lead to the same conclusion in comparing different methods’ performance under camera jitter, and, again, *NMSE* is more sensitive. Due to the failure of tracking feature points after camera jitter, the OF method produces no values for both indicators. Moreover, for the STC method, the target mistracking caused by substantial camera jitter even leads to a negative value of *NMSE*, much smaller than the positive ρ. Besides, when compared with Scenario I, *NMSE* varies from 0.987 to no value for LK, from 0.988 to negative value for STC-1, and from 0.992 to 0.925 for MSTC-1. The minimum loss of *NMSE* indicates the best performance of the MSTC method in resisting the interference of camera jitter.
(3) A significant influence on the measurement results similar to Scenarios I-III still holds for the frame rate of the smartphone. See the MSTC-1 measurement, for example, due to inadequate choice of 60 FPS instead of 59.96 FPS, *NMSE* drops from 0.925 to 0.894 and ρ from 0.964 to 0.948.
(4) The MSTC method, as compared to the STC method, shows slightly better performance in measuring target 2 and much better performance in measuring target 1, proving once again a better anti-interference ability of the proposed method. See also in Table 6, *NMSE* takes 0.933 and 0.926 for MSTC-2 and STC-2, respectively, while 0.925 and negative value for MSTC-1 and STC-1, respectively.
(5) Deviations of the MSTC method are smaller than those of the STC method, in which the target 2 shows better performance than the target 1.

### 7.3. Computation Efficiency

After the iPhone 7 recorded the videos of sphere vibration in four scenarios, we implemented all the above displacement identification with Matlab codes on a laptop computer, which is of moderate hardware configuration, e.g., Intel Core i5-4200H 2.8GHz and 4G memory.

During the identification, each 15-s vibration video with 60 FPS contains 900 frame images to be analyzed, among which the first one is shown in Figure 13. The time consumed for the identification with different vision methods in four scenarios is listed in Table 7.

It can be seen from Table 7 that the MSTC method takes 0.14s~0.18s to identify the displacement of one target between two adjacent frame images. Comparatively, the STC method and the OF method take 0.12~0.16 s and 0.08~0.10 s, respectively. Despite a slight increment, i.e., 0.02 s introduced by additional calculation of the context influence matrix, the MSTC method is still applicable for the design of online measurement via its high efficiency, similarly to the STC method that has seen online tracking practice.

### 7.4. Summary

To sum up, several conclusions can be drawn from all the above observations,

(1) In the vision-based measurement of engineering vibration, the actual frame rate, instead of the theoretical one of the camera, is crucial to achieving a high-accuracy result of displacement time history.
(2) To evaluate the performance of vision methods, the indicator, *NMSE*, is more critical than ρ when interference is involved.
(3) In an interference environment, the OF method is prone to mismatch the feature points and lead to data deviated or lost. The conventional STC method is sensitive to target selection and can effectively track those targets having a large proportion of pixels in the context with motion tendency similar to the target center. The proposed MSTC method, however, can ease the sensitivity to target selection through in-depth processing of the information in the context and finally enhance the robustness of the target tracking.
(4) The OF method has the highest computation efficiency in measuring target displacement, followed by the STC method and the MSTC method. Although the MSTC method requires nearly 10% more time than the STC method to measure displacement, the average time cost consumed for tracking one target between adjacent frame images is far less than 1 s, favoring its potential application in online measurement.

## 8. Further Discussion

In the present study, we develop an improved vision method for structural displacement measurement. This method uses a smartphone to perform multi-point displacement measurements in environments with illumination variation, fog interference, and camera jitter. Despite the high-accuracy results, there still exist two issues to be addressed:

(1) Temporal aliasing: Compared with professional high-speed cameras, smartphone cameras have a lower sampling frequency, i.e., frame rate, for video shooting, mostly 30/60 FPS, which is vulnerable to time aliasing. According to the Nyquist sampling theorem, the sampling frequency of measurement should be at least two times the highest frequency of response to avoid high-frequency aliasing information. In wired or wireless sensor systems, anti-aliasing filters are usually used to eliminate aliasing effects. In vision systems, however, temporal aliasing cannot be removed because the image has already been aliased when it is taken. Therefore, when the Nyquist sampling theorem cannot be satisfied with the smartphone, a professional camera with a higher frame rate has to be applied for accurate measurement.
(2) Generalized camera motion: In the experiment, the present method is verified effective under camera jitter, and target displacements can be obtained through directly subtracting the measurement of a non-moving reference point from the overall measurements. In engineering practice, however, the camera motion can be more generalized and complex, such as that of a UAV-carried camera, and reference points cannot fully provide 6-DOF (degrees-of-freedom) camera motions. In this case, to measure the dynamic displacements, it is necessary to introduce new technologies such as dual-camera based motion compensation [28] to exclude the influence of camera motion.

## 9. Conclusions

In this paper, we studied the application of computer vision technology in the field of structural vibration. A multi-target monitoring method based on a motion-enhanced spatio-temporal context (MSTC) algorithm developed herein was proposed, which realizes the synchronous measurement of multi-point dynamic displacement in an interference environment by using smartphone-recording video. A series of sine-sweep vibration experiments considering different interference factors were carried out to investigate the performance of the proposed method. Test results show good consistency in different scenarios, indicating that the proposed method preserves good adaptability to complex backgrounds and strong resistance to illumination variation, fog interference, and camera jitter. Besides, the proposed method acts insensitive in selecting the target region and efficient in tracking targets, which are beneficial to potential engineering applications.

Although the designed experiments have involved several interference factors, it is no doubt that, for comprehensive performance validation, more investigations on other objects of various profiles and textures are still required, as well as field tests on real vibrating structures. These ongoing validations and further studies on more complex environment changes, such as occlusion, strong wind, and rain, are crucial to extending the vision-related applications.

## Figures and Tables

**Table 1:** Intrinsic parameters of iPhone 7 back camera.

Parameter	$f_{x}$	$f_{y}$	θ	$x_{0}$	$y_{0}$
value	3322.5	3322.3	1.6869	2010.3	1480.6

**Table 2:** Distortion coefficients of iPhone 7 back camera.

Coefficient	$k_{1}$	$k_{2}$	$p_{1}$	$p_{2}$
value	0.2999	−1.6605	3.03 × 10^−4^	3.80 × 10^−3^

**Table 3:** Evaluation of displacement results without interference factors.

	59.96 FPS							
	*NMSE*	ρ	Maximum Displacement			Minimum Displacement		
			Vision (mm)	Sensor (mm)	Deviation	Vision (mm)	Sensor (mm)	Deviation
MSTC-1	0.992	0.996	5.768	5.464	5.55%	−5.405	−5.495	−1.65%
MSTC-2	0.992	0.996	5.381		−1.52%	−5.961		8.48%
STC-1	0.988	0.995	5.379		−1.56%	−6.071		10.48%
STC-2	0.991	0.996	5.649		3.37%	−5.994		9.09%
LK	0.987	0.994	5.460		−0.09%	−6.266		14.03%
	60FPS							
MSTC-1	0.953	0.977	5.768	5.900	−2.24%	−5.405	−5.938	−8.98%
MSTC-2	0.966	0.984	5.381		−8.79%	−5.961		0.39%
STC-1	0.948	0.975	5.379		−8.82%	−6.071		2.24%
STC-2	0.960	0.983	5.649		−4.26%	−5.994		0.95%
LK	0.935	0.969	5.460		−7.46%	−6.266		5.53%

**Table 4:** Evaluation of displacement results with illumination variation.

	59.96 FPS							
	*NMSE*	ρ	Maximum Displacement			Minimum Displacement		
			Vision (mm)	Sensor (mm)	Deviation	Vision (mm)	Sensor (mm)	Deviation
MSTC-1	0.977	0.989	5.277	5.758	−8.35%	−6.000	−5.780	3.81%
MSTC-2	0.988	0.994	5.555		−3.53%	−5.620		−2.76%
STC-1	0.957	0.988	4.821		−16.27%	−6.600		14.19%
STC-2	0.987	0.994	5.524		−4.06%	−6.000		3.81%
LK	0.778	0.968	6.948		20.67%	−4.569		−20.95%
	60 FPS							
MSTC-1	0.885	0.945	5.277	5.679	−7.09%	−6.000	−5.550	8.12%
MSTC-2	0.903	0.953	5.555		−2.20%	−5.620		1.27%
STC-1	0.865	0.941	4.821		−15.12%	−6.600		18.93%
STC-2	0.901	0.952	5.524		−2.73%	−6.000		8.12%
LK	0.652	0.908	6.948		22.33%	−4.569		−17.67%

**Table 5:** Evaluation of displacement monitoring results with fog interference.

	59.96 FPS							
	*NMSE*	ρ	Maximum Displacement			Minimum Displacement		
			Vision (mm)	Sensor (mm)	Deviation	Vision (mm)	Sensor (mm)	Deviation
MSTC-1	0.969	0.986	5.602	5.453	2.75%	−6.050	−5.528	9.45%
MSTC-2	0.972	0.990	5.745		5.36%	−6.000		8.54%
STC-1	0.948	0.984	5.667		3.93%	−6.800		23.01%
STC-2	0.971	0.988	5.787		6.13%	−6.150		11.26%
LK	0.501	0.917	10.129		85.76%	−4.562		−17.48%
	60 FPS							
MSTC-1	0.872	0.939	5.602	5.515	1.58%	−6.150	−5.557	10.66%
MSTC-2	0.888	0.945	5.745		4.17%	−6.000		7.96%
STC-1	0.850	0.937	5.667		2.75%	−6.800		22.36%
STC-2	0.882	0.944	5.787		4.93%	−6.150		10.66%
LK	0.413	0.875	10.129		83.65%	−4.562		−17.92%

**Table 6:** Evaluation of displacement monitoring results with camera jitter.

	59.96 FPS							
	*NMSE*	ρ	Maximum Displacement			Minimum Displacement		
			Vision (mm)	Sensor (mm)	Deviation	Vision (mm)	Sensor (mm)	Deviation
MSTC-1	0.925	0.964	5.286	5.868	−9.92%	−5.643	−5.860	−3.71%
MSTC-2	0.933	0.967	5.514		−6.03%	−5.808		−0.89%
STC-1	−29.227	0.156	29.796		407.79%	−19.659		235.47%
STC-2	0.926	0.965	5.400		−7.97%	−6.081		3.77%
LK	/	/	/		/	/		/
	60 FPS							
MSTC-1	0.894	0.948	5.186	5.840	−11.20%	−5.643	−5.742	−1.73%
MSTC-2	0.900	0.950	5.514		−5.58%	−5.808		1.15%
STC-1	−36.539	0.142	29.796		410.22%	−19.659		242.36%
STC-2	0.887	0.945	5.400		−7.53%	−6.081		5.90%
LK	/	/	/		/	/		/

**Table 7:** Time consumption for displacement identification.

		Total Number of Targets	Total Number of Frame Images	Total Time Cost (s)	Average Time Cost (s/Target/Frame)
	MSTC	2	900	258	0.143
Scenario I	STC	2	900	230	0.128
	LK	1	900	79	0.088
	MSTC	2	900	262	0.146
Scenario II	STC	2	900	232	0.129
	LK	1	900	84	0.093
	MSTC	2	900	268	0.149
Scenario III	STC	2	900	235	0.131
	LK	1	900	85	0.094
	MSTC	3	900	472	0.175
Scenario IV	STC	3	900	416	0.154
	LK	1	/	/	/
